# Global action to reduce HIV stigma and discrimination

**DOI:** 10.7448/IAS.16.3.18881

**Published:** 2013-11-13

**Authors:** Cynthia I Grossman, Anne L Stangl

**Affiliations:** 1 Division of AIDS Research, National Institute of Mental Health, Bethesda, MD, USA; 2 Department of Global Health, International Center for Research on Women, Washington, DC, USA; 3 Secretariat, Stigma Action Network, Washington, DC, USA

**Keywords:** HIV, AIDS, stigma, discrimination, interventions, key populations

## Abstract

There is no question that the stigma and discrimination associated with HIV and AIDS can be reduced through intervention. The inclusion of stigma and discrimination reduction as a critical component of achieving an AIDS-free generation in recent UNAIDS, UN and PEPFAR political initiatives is promising. Yet national governments need evidence on effective interventions at the individual, community and societal levels in order to strategically incorporate stigma and discrimination reduction into national AIDS plans. Currently, the heterogeneity of stigma and discrimination reduction approaches and measurement makes it challenging to compare and contrast evaluated interventions. Moving forward, it is critical for the research community to: (1) clearly link intervention activities to the domains of stigma to be shifted; (2) assess the stigma domains in a consistent manner; and (3) link stigma and discrimination reduction with HIV prevention, care and treatment outcomes (e.g., uptake, adherence and retention of ART). These steps would further advance the scientific evidence base of stigma and discrimination reduction and allow for the identification of effective interventions that could be scaled up by national governments.

## Stigma and discrimination reduction: a critical component of achieving an AIDS-free generation

Given recent advances in biomedical prevention, the global community has begun to seriously contemplate an AIDS-free generation for the first time in three decades. The Joint United Nations Programme on HIV/AIDS (UNAIDS) outlined a vision for “getting to zero” in its strategic plan for 2011–2015, including zero new infections, zero AIDS-related deaths and zero discrimination [1]. National governments are now being encouraged and supported to scale up evidence-based, efficacious HIV prevention and treatment technologies [2] in order to achieve the ambitious goals agreed to by UN member states at the 2011 UN high-level meeting on HIV/AIDS [3]. The President's Emergency Plan For AIDS Relief (PEPFAR) Blueprint, which proposes critical steps needed to achieve an AIDS-free generation, was launched in 2012 [4]. A common element of these political initiatives is their recognition that reducing HIV-related stigma and discrimination is critical to the success of the global HIV response.

The field of stigma research related to HIV has advanced rapidly, and while many questions remain unanswered and gaps in empirically derived data exist, there is no question that stigma can be reduced [5, 6]. Specific research strides include a solid evidence base of valid measures that capture multiple domains of stigma associated with HIV [7, 8], including one measure showcased in this special issue. Although there remains a need for empirically derived data, stigma and discrimination interventions have been developed and implemented in the field [9, 10], as well as a comprehensive toolkit of stigma reduction activities [11], components of which are being tested as part of National Institutes of Health-supported research [12]. The healthcare sector has one of the strongest evidence bases regarding stigma and discrimination measurement and intervention [13–15]. Beyond the healthcare setting, addressing stigma among the general community has been a focus for research, but to varying degrees of success [6]. Much of the work has included community education campaigns associated with HIV testing, including some community mobilization strategies. With regard to stigma measurement among people living with HIV (PLHIV), several measures have been developed [16–18], with the PLHIV Stigma Index serving as both an assessment and a community engagement and empowerment tool [19]. Despite these strides, the absence of evidence demonstrating a clear link between stigma and discrimination reduction and HIV outcomes, as well as the cost-effectiveness of these approaches, is impeding their prioritization by implementing organizations, ministries of health and other entities.

## The price of inaction: the impact of stigma and discrimination on HIV prevention and care programmes

The persistence of HIV-related stigma and discrimination is evident in research and programmatic data alike, despite treatment advances that have turned HIV into a chronic, manageable condition. Thirty years into the HIV pandemic, stigma and discrimination continue to impede individuals and communities from accessing and benefiting from effective prevention and treatment strategies. There is mounting evidence that HIV-related stigma and discrimination are barriers to HIV testing [20], sero-status disclosure [21], retention in care [22] and uptake of and adherence to antiretroviral therapy (ART) [23, 24]. There is also evidence of the associations between HIV-related stigma and racism, poverty and heterosexism, although the complexities of these associations and interactions are only beginning to be unravelled via research [25–27]. In many settings, the stigma associated with HIV is fuelled by laws and policies that keep key populations at risk of HIV infection and PLHIV at the margins of society, despite evidence of the negative public health impact of criminalization [28, 29].

Included among these discriminatory laws are those that make illegal lesbian, gay and bisexual relationships; expressing one's transgender identity; drug use; and sex work, and those that create barriers to legal protections for these groups and for young people, women and migrants. At least 47 countries have used the criminal law to prosecute PLHIV for non-disclosure of their HIV status, potential exposure of others to HIV, or transmission of HIV, regardless of whether there was any intent to transmit, harm reduction measures were adopted, the person with HIV risked violence if she or he disclosed, or transmission actually occurred [30]. Many of these laws provide for criminal prosecution of PLHIV for behaviours that bear little to no risk of transmission, such as for spitting or biting [31]. Yet, as highlighted in the review by Stangl et al. [32] in this special issue, no interventions to reduce HIV-related discrimination have been assessed in the peer-reviewed or grey literature, and very few intervention tools exist for reducing intersecting HIV and key population stigmas [33, 34].

The fact that stigma associated with HIV continues to hamper prevention and treatment efforts is particularly distressing given the unprecedented number of effective tools available, including recent advances such as voluntary medical male circumcision, pre-exposure prophylaxis and ART for the purposes of extending the lives of PLHIV and providing HIV prevention benefits for their sexual partners in the context of viral suppression [5–37]. In addition, many countries are rolling out Option B+, which provides ART to expectant mothers living with HIV for their lifetime, regardless of CD4 cell count, as opposed to a shorter course around pregnancy, childbirth and breastfeeding [38]. These tools are in various stages of being brought to scale, but as the data on the role of HIV stigma as a barrier to testing, retention in care and treatment suggest, these tools may not reach their full potential if the stigma and discrimination associated with HIV remain unaddressed.

For example, in 2011 UNAIDS estimated that 46% of people eligible for ART in low- and middle-income countries did not receive it [39]. In countries where all pregnant women presenting for prenatal care are tested for HIV and provided treatment access, there is still an alarmingly high rate of women who refuse to present for prenatal care due to the stigma associated with HIV, despite access to effective treatment [20, 40]. As certain populations fail to access, or in some cases are excluded from accessing, resources, such as quality healthcare, food, housing and employment opportunities, based on factors other than HIV status (e.g., race or sexual orientation), the stigma associated with HIV acts in a compounding fashion to further exacerbate disparities [8, 41, 42].

Despite the advances in HIV stigma research over the last decade that are mentioned here, the gap in the evidence base on effective interventions is hampering national governments from integrating stigma and discrimination reduction – critical enablers of the HIV response – into national AIDS plans, and is threatening our collective ability to get to zero. As national governments seek to bring HIV prevention and treatment to a larger scale amidst multiple resource constraints, both human and financial, there needs to be strong evidence for the impact of stigma reduction efforts and high-quality data that can inform evidence-based decision making around priorities at the national level. To respond to this gap in the evidence base, a redoubling of research efforts to reduce stigma and discrimination across a variety of settings and within all populations is needed. The goal of this special issue is to enhance the peer-reviewed literature with strong, scientifically sound evidence for stigma reduction interventions. The intention is also to encourage the research field to consider additional ways to reduce stigma and discrimination, acknowledge and address the challenges with research methodology and create a sense of timeliness and urgency for the development and testing of stigma and discrimination reduction efforts.

## A common conceptualization of HIV-related stigma and discrimination: critical for generating evidence

Currently, the heterogeneity of stigma and discrimination reduction approaches and measurement makes it challenging to compare and contrast evaluated interventions, as evidenced by the lack of meta-analyses of stigma reduction interventions in the literature. While there is general agreement around four intervention categories originally described by Brown *et al*. [5] (i.e., information-based approaches, skills building, counselling and support, and contact with affected groups), there is less agreement about how to measure the success of these approaches at influencing the various domains of HIV stigma. This stems from the lack of a common conceptualization of the stigmatization process that can inform research, programmatic and evaluation efforts.

The foundation of HIV stigma research is Erving Goffman's seminal conceptualization of stigma as a discrediting attribute that creates a “spoiled identity,” which cuts the stigmatized person “off from society and from himself” [43]. More recent conceptualizations have highlighted the societal and structural nature of stigma, and have attempted to articulate the process of stigmatization [44, 45] and distinguish stigma from discrimination [46]. These conceptualizations have framed current understanding regarding the need to intervene at multiple socio-ecological levels (i.e. individual, interpersonal, organizational, community and public policy) to reduce HIV-related stigma and discrimination [47]. A recent global effort to develop standardized indicators of HIV stigma and discrimination led to the development of a practical framework to inform stigma reduction programming and measurement [48]. This framework defines specific domains, including drivers, facilitators, intersecting stigmas and manifestations of stigma, that can be shifted through programmatic efforts, and it proposes measures to assess each domain [49]. Moving forward, it is critical for the research community to (1) clearly link intervention activities to the domains of stigma to be shifted; (2) assess the stigma domains in a consistent manner; and (3) link stigma and discrimination reduction with HIV prevention, care and treatment outcomes (e.g., uptake, adherence and retention of ART). These steps would further advance the scientific evidence base of stigma and discrimination reduction and allow for the identification of effective interventions that could be scaled up by national governments.

## Global action to reduce stigma and discrimination

Given the importance of reducing HIV-related stigma and discrimination for realizing an AIDS-free generation, this special issue takes stock of current strategies for interrupting the stigmatization process, reducing the negative manifestations of stigma and discrimination, and creating an enabling environment for HIV prevention, care and treatment strategies. The articles in this issue review and organize current evidence on approaches for reducing stigma and discrimination in the healthcare setting, among the general public and among PLHIV and key populations. They draw attention to methodological and measurement challenges in evaluating stigma and discrimination reduction interventions, highlight innovative approaches for addressing stigma in a variety of populations and contexts, and identify critical gaps in these approaches that must be addressed in future research. They also provide insights into the determinants of key population stigmas to inform future intervention development to address intersecting stigmas.

In addition to academic peer reviews, all of the manuscripts benefitted from careful review by individuals directly impacted by stigma and discrimination, via a panel of reviewers from communities of PLHIV and members of key population groups. These reviews were critical to ensuring attention to terminology and the relevance of findings to programmatic efforts, and for clarifying ways in which the research would benefit individuals and communities while simultaneously advancing the science.

Several key issues are highlighted in this supplement. First, the review articles reinforce the need for effective stigma and discrimination reduction interventions that can be taken to a national-level scale, and they identify key gaps in current HIV stigma research and methodology that require intensified efforts. The review by Katz et al. [50] synthesizes the evidence for the link between stigma and adherence across a number of studies. As adherence is critical for PLHIV to achieve viral suppression and benefit fully from the individual and prevention benefits of ART, the link between stigma and adherence provides a sobering picture of the work left to do to achieve the full benefits of universal access to ART. The review by Stangl et al. [32] synthesizes evaluation data from nearly 50 interventions, documenting the considerable progress made over the past decade and identifying key gaps and impediments to the identification of effective stigma reduction strategies, including the heterogeneity of measures used to assess stigma domains, the paucity of interventions designed to address multiple sociological levels concurrently and the lack of studies comparing the effectiveness of different stigma reduction strategies and studies assessing the influence of stigma reduction on key behavioural and biomedical outcomes.

Second, at the same time that they are negatively impacted by stigma and discrimination, PLHIV are critical for the success of stigma reduction interventions. In particular, group-based approaches led by or actively involving PLHIV hold promise for responding to HIV-related stigma and discrimination at the community level. For example, an intervention in Uganda found that groups of PLHIV working collectively to reduce stigma and discrimination in their communities bolstered confidence among members, reduced self-stigma and improved group members’ ability to deal with external HIV stigma when encountered [51]. Likewise, an intervention in Thailand that paired business partners living with HIV with those who were HIV negative, and trained them to engage their communities in stigma reduction activities, appears to have led to community-level reductions in fear of HIV infection and shame associated with HIV [52].

Third, this supplement reflects the substantial progress that has been made towards reducing stigma in healthcare settings. Efficacious health facility-based [53] and medical school-based interventions [54] now exist to reduce stigma and discrimination towards PLHIV, and a standardized tool for assessing HIV-related stigma in health facilities [55] has been developed. In addition, this special issue contains the first ever evaluation of a discrimination reduction intervention, which integrated legal literacy and legal services into health facilities in Kenya. Findings suggest that legal empowerment programmes have the potential to improve access to justice and health among marginalized groups (including PLHIV), promote accountability among healthcare providers and contribute to altering unjust structures and systems [56]. These advances are timely, given the need to take effective strategies to scale, as evidenced by PLHIV Stigma Index data, which highlight how commonplace it is globally to experience stigma in healthcare settings [19, 57], and an article in this special issue that found high levels of stigma in urban health facilities in India. For example, providers expressed a willingness to prohibit women living with HIV from having children (55–80%), endorsed mandatory testing for female sex workers (94–97%) and surgery patients (90–99%) and stated that people who acquired HIV through sex or drugs “got what they deserved” (50–83%) [58].

Finally, this special issue presents new evidence to inform the development of interventions to reduce stigma towards key populations, specifically men who have sex with men, people who inject drugs and African Americans. This set of manuscripts highlights the need to integrate stigma reduction with HIV prevention messages and activities and the importance of investigating the impacts of the larger socio-political and economic contexts on stigma and healthcare utilization. Two articles have attempted to expand the reach of stigma reduction and HIV prevention for key populations in the United States, one via African American churches and one via the internet. Berkley-Patton et al. [59] piloted an intervention to increase HIV awareness and testing among members of African American churches. While attitudes and willingness to receive an HIV test improved over the course of the intervention, stigma remained unchanged. The reasons for the lack of change in stigma and the implications for HIV prevention uptake remain worthy of further investigation. Christensen et al. [60] found that a journal-based game was successful at reducing shame and sexual risk-taking behaviour among young men who have sex with men in the United States. Such technology-based interventions have the potential to greatly expand the reach of both stigma reduction and HIV prevention messages to young men who have sex with men in contexts with widespread access to the internet.

In contexts where same-sex behaviour is criminalized, different types of interventions may be needed to address both the stigma experienced by men who have sex with men and the discriminatory laws and policies that fuel the stigmatization process. Risher et al. [61] found high levels of stigma among men who have sex with men in Swaziland (i.e., 61.7% feared seeking healthcare, 44.1% experienced some form of stigma and 73.9% perceived social stigma from family and friends) and identified a number of factors associated with non-disclosure of sexual behaviour to healthcare providers and fear of seeking healthcare, including having experienced legal discrimination as a result of one's sexual orientation or practices. The analysis provides several insights for developing structural interventions to increase healthcare seeking and disclosure of sexual practices to healthcare workers and facilitate behavioural and biomedical HIV prevention approaches among men who have sex with men in Swaziland.

Also relevant to developing structural interventions, the article by Lim et al. [62] demonstrates the importance of considering education and income inequality in designing interventions to reduce stigma towards people who inject drugs in Viet Nam. The analysis revealed that individual-level educational attainment was significantly associated with less stigmatizing attitudes, and this relationship superseded community-level inequalities in education and income.

Research, policy and programmes that seek to address the HIV epidemic are in unprecedented alignment in their call to scale up the tools to bring about an AIDS-free generation. As evidenced by the foreword to this special issue, key agencies are also in alignment regarding the importance of stigma and discrimination reduction and its role in facilitating scale-up and uptake of HIV prevention, care and treatment. This supplement is as important for the advances that it highlights as well as the gaps it identifies. As the evidence base grows, so too will the ability of national governments to make data-driven decisions about scaling up stigma and discrimination reduction efforts. It is incumbent on the research community to provide data that will help governments make efficient and effective use of resources spent on stigma and discrimination reduction. That said, it is important to recognize that all programmatic efforts take resources, evidence and political will. This supplement is the start of a discussion regarding the evidence for stigma and discrimination reduction efforts. It is also a call to action for even more refined research activities, for greater community involvement (particularly of key populations in research and programmatic efforts) and for scale-up of some programmatic principles that have been identified, while including high-quality monitoring and evaluation strategies to further expand the evidence base. It is our hope that within the next decade, cost-effective interventions will be identified and countries will be collecting programmatic data demonstrating the impact of stigma and discrimination reduction on HIV prevention and care outcomes.
